# Clinical Guideline for Microwave Ablation of Bone Tumors in Extremities

**DOI:** 10.1111/os.12749

**Published:** 2020-08-09

**Authors:** Kai Zheng, Xiuchun Yu, Yongcheng Hu, Yingze Zhang, Zhen Wang, Sujia Wu, Jingnan Shen, Zhaoming Ye, Chongqi Tu, Yu Zhang, Xing Wei, Yong Hu, Xuquan Wang, Jiazhen Li, Hong Duan, Yuxian Wu, Ming Xu, Zhenchao Yuan, Yongzhong Wei, Bingyao Chen

**Affiliations:** ^1^ Department of Orthopedics The 960th Hospital of the PLA Joint Logistice Support Force Jinan China; ^2^ Department of Bone Oncology Tianjin Hospital Tianjin China; ^3^ Department of Orthopaedic Surgery The Third Hospital of Hebei Medical University, Orthopaedic Research Institute of Hebei Province Shijiazhuang China; ^4^ Department of Bone Oncology, Xijing Hospital Air Force Military Medical University Xi'an China; ^5^ Department of Orthopedics, School of Medicine, Jinling Hospital Nanjing University Nanjing China; ^6^ Department of Bone Oncology The First Affiliated Hospital of Sun Yat Sen University Guangzhou China; ^7^ Department of Orthopaedics, The Second Affiliated Hospital Zhejiang University School of Medicine Hangzhou China; ^8^ Department of Orthopedics, West China Hospital Sichuan University Chengdu China; ^9^ Department of Orthopedics, Guangdong Provincial People's Hospital Guangdong Academy of Medical Sciences Guangzhou China; ^10^ Department of Orthopedics Aerospace Center Hospital Beijing China; ^11^ Department of Bone Disease and Bone Tumors Surgery, The First Affiliated Hospital Anhui Medical University Hefei China; ^12^ Department of Orthopedics Guiqian International General Hospital Guiyang China; ^13^ Department of Orthopaedics The First Affiliated Hospital of Zhengzhou University Zhengzhou China; ^14^ Department of Bone Oncology, Qilu Hospital (Qingdao), Cheeloo College of Medicine Shandong University Qingdao China; ^15^ Department of Bone and Soft Tissue Surgery Guangxi Medical University Cancer Hospital Nanning China; ^16^ Department of Orthopaedics The First Affiliated Hospital With Nanjing Medical University Nanjing China

**Keywords:** Ablation Techniques, Bone Neoplasms, Extremities, Guideline, Microwave

## Abstract

Microwave ablation has been used to treat bone tumors in extremities for more than 30 years. With improved recognition, updated microwave equipment, and expanded clinical application, microwave ablation has recently been widely used to treat bone tumors. To standardize the application of microwave ablation in the clinical treatment of bone tumors in the limbs, research results and clinical experience involving the use of microwave ablation to treat bone tumors in the limbs have been summarized, and a clinical guideline has been designed. This guideline is aimed at providing a reliable clinical basis for indications, preoperative evaluation and decision‐making, perioperative treatment, complications, and other issues *via* evidence‐based medicine. Two aspects are considered—percutaneous microwave ablation and intraoperative microwave ablation of bone tumors in extremities. Ultimately, the guideline is intended to standardize treatment and improve the clinical efficacy of microwave ablation of bone tumors in extremities.

Microwave ablation is a commonly used method in tumor ablation. The technique involves the application of the thermal effect of a microwave electromagnetic field to inactivate the tumor, including directly killing the tumor^1^, inducing apoptosis^2^, ^3^, destroying tumor blood vessels^4^, and promoting immunity^5^, ^6^, ^7^, ^8^, among others. Microwave ablation has been used for many years in the treatment of solid organ tumors, which proves that this technique has a certain clinical application value^9^, ^10^, ^11^. As hard tissue, the main components (collagen and inorganic salt) of the bone tissue can withstand elevated temperatures, and the maintenance of the biomechanical strength is enhanced. Thus, microwave ablation presents distinct advantages and characteristics for the treatment of bone tumors^12^.

Microwave ablation has been used in the treatment of bone tumors for more than 30 years. In its development, progress mainly includes the following aspects. First, the equipment has been improved. Compared with the previous equipment, the microwave ablation radiator is more flexible and efficient, the temperature measurement system is more rapid and accurate, and the degree of automation is more intelligent. Second, the scope of application has expanded. From the initial stage of treating primary malignant bone tumors, the treatment has also been used to treat metastatic bone tumors, malignant soft tissue tumors, and benign bone tumors; moreover, the operation and operating technology have become increasingly standardized. Microwave ablation can be used as an independent percutaneous minimally invasive treatment for some benign bone tumors and bone metastases^13^, ^14^, or as an auxiliary treatment for hemostasis, tumor inactivation or improving the safety of tumor resection boundary^15^, ^16^, ^17^, ^18^, ^19^.

The clinical application of microwave ablation in the treatment of bone tumors in extremities has gradually improved, and new research and application have successfully been reported. This guideline is formulated from evidence‐based clinical data, experiences of Chinese experts, and the current situation in China. The clinical guideline for microwave ablation of bone tumors in extremities includes recommendations 1–6 for percutaneous microwave ablation of bone tumors in extremities, 7–20 for intraoperative microwave ablation of bone tumors in extremities, and 21–22 for product use, including evaluation and selection before microwave ablation, operation during microwave ablation, complications of microwave ablation, and so on. This study is intended to provide a reliable clinical basis for microwave ablation of bone tumors in extremities, standardize treatment, and improve the therapeutic effect of the technique.

## Recommendation 1: Percutaneous Microwave Ablation of Osteoid Osteoma in Extremities is Effective

Osteoid osteoma is a common benign bone tumor in extremities with unknown etiology, often accompanied by nocturnal pain. The cortex of a long bone is prone to develop the tumor. Osteoid osteoma typically consists of tumor nests at the center and thickened bone around. Treatment of tumor nests is the fundamental method of treatment^20^, ^21^, ^22^. A simple systematic literature review was conducted for percutaneous microwave ablation of an osteoid osteoma in extremities. The femur (41 cases) and tibia (20 cases) are the most common surgical sites, as determined from 76 patients with an average age of 20.6 years (range, 3–48 years). The average tumor size was 6.8 mm, and the average follow‐up time was 17.9 months. Two patients reported postoperative recurrence. Moreover, six patients suffered operative complications (7.9%), including three cases of skin numbness, one case of puncture site infection, one case of radial nerve injury and two‐stage skin burn, and one case of three‐stage skin burn^13^, ^14^, ^23^, ^24^, ^25^, ^26^. More patients with osteoid osteoma in extremities have been treated by percutaneous radiofrequency ablation^27^, ^28^. The new view is that microwave ablation of osteoid osteoma provides fast heating and short ablation time, avoids skin burns caused by electrode pieces, and is not affected by pacemakers, among other factors. Owing to these characteristics, microwave ablation is a preferred choice for the percutaneous treatment of osteoid osteoma^14^, ^23^.

Percutaneous microwave ablation of osteoid osteoma has the advantages of high effective rate, low recurrence rate, and few complications. The risk of puncture and microwave ablation can be assessed by preoperative imaging, and the puncture route of an important tissue structure can be avoided. Microwave ablation can be performed again on patients with recurrence.

## Recommendation 2: Microwave Ablation is a Choice for Patients with Bone Metastases in Extremities That is Resistant to Traditional Treatments

Long bones of extremities are common sites of bone metastasis. Epidemiological studies have found that 48.9% of bone metastases occur in the long bones of the limbs^29^. In addition to surgery, radiotherapy, and drug treatment, percutaneous microwave ablation can effectively relieve pain in the treatment of limb bone metastasis. Combined with bone cement filling, this method can enhance bone strength. Leakage of bone cement is a common complication^30^, ^31^, ^32^. Evident pain symptoms of bone metastasis in extremities, localized metastasis lesions, and a safe puncture path are considered good indications for percutaneous microwave ablation^33^. Percutaneous ablation of malignant bone tumors is also only suitable for tumors with slow growth and requires two typical characteristics: fewer than three tumor sites, with the largest tumor diameter being less than 3 cm^34^.

Patients with bone metastases in extremities experience obvious pain, identify the exact location of pain, and cannot tolerate the routine operation. The risks and benefits of microwave ablation can be evaluated with the whole body and can consider the local bone strength of patients. Percutaneous microwave ablation can be used to treat bone metastases in extremities to alleviate the pain. After ablation, pathological fractures may occur. Ablation can be combined with percutaneous internal fixation if necessary.

## Recommendation 3: Percutaneous Microwave Ablation of Bone Tumors in Extremities Should be Guided by Imaging

Computer tomography (CT) is the most commonly used imaging method to guide percutaneous microwave ablation of bone tumors in extremities^13^, ^14^, ^23^, ^25^, ^26^, ^30^, ^31^. In a systematic literature analysis of 76 patients with an osteoid osteoma in extremities treated by imaging‐guided percutaneous microwave ablation, 52 patients were treated with guidance of conventional CT, and the remaining 24 patients were treated by microwave ablation guided by cone‐beam CT combined with intraoperative navigation. No significant difference in treatment efficiency was determined between the two groups^13^, ^14^, ^23^, ^24^, ^25^, ^26^. No significant differences in technical success rate, clinical success rate, and complication rate were found between CT and cone‐beam CT. The radiation dose received by the cone‐beam CT group was significantly less than that received by the conventional CT Group^35^. Positron emission tomography–computed tomography (PET‐CT) is also used to guide puncture and percutaneous microwave ablation, which helps to improve puncture accuracy^36^.

Percutaneous microwave ablation of bone tumors in extremities requires imaging guidance, which can be provided by CT, cone‐beam CT, magnetic resonance imaging (MRI), and PET‐CT to varying levels. Intraoperative navigation technology and robot‐assisted technology can improve puncture accuracy and safety.

## Recommendation 4: Percutaneous Microwave Ablation of Osteoid Osteoma in Extremities Should be Performed Under Appropriate Anesthesia

Although percutaneous microwave ablation of tumors is a minimally invasive operation, it releases high‐energy thermal radiation during surgery^37^, causing severe pain, particularly pain from osteoid osteoma tumor nest stimulation; local anesthesia typically fails to meet surgical requirements^38^. In addition, high‐energy heat treatment may induce increases in body temperature and basic metabolic rate, which need strict anesthesia management. Patients with poor coordination can choose general anesthesia^37^, whereas patients with good coordination can choose regional block anesthesia and sedation^13^. Systematic literature analysis of 76 patients with an osteoid osteoma in extremities, 55 cases of general anesthesia, eight cases of epidural anesthesia, seven cases of epidural anesthesia combined with intravenous sedation and analgesia, five cases of local anesthesia combined with intravenous sedation and analgesia, and one case of local anesthesia only (the patient received insufficient ablation and suffered tumor recurrence because of the server operative pain)^13^, ^14^, ^23^, ^24^, ^25^, ^26^.

Percutaneous microwave ablation of osteoid osteoma in extremities often induces severe pain in patients. Puncture and percutaneous microwave ablation should be performed under general anesthesia. During ablation, patients may exhibit pain reaction because of the depth of insufficient anesthesia, which may lead to a change in microwave needle position and thermal injuries in other tissues.

## Recommendation 5: The Power and Time of Percutaneous Microwave Ablation of Bone Tumors in Extremities Should be Determined Based on Different Equipment Settings

The frequencies of medical microwave ablation include 915 and 2450 MHz. The 2450 MHz frequency has been widely used because of its high power, fast heat production, and good ablation form. The difference in microwave output power, loss, and microwave needle design can lead to a difference in the range of microwave ablation^39^. In the systematic literature review of percutaneous microwave ablation of osteoid osteoma in extremities, 76 patients received microwave ablation equipment from six companies for ablation. Despite variations in ablation parameters, good clinical efficacy was observed in all patients: seven patients received microwave ablation at 20 W for 2 min; 24 patients received microwave ablation at 30 W for 30 s pause (three cycles), and the target temperature was 90°C (otherwise, the number of cycles was increased); 13 patients received microwave ablation at 50–60 W for 1–2.5 min; 24 patients received 915 Hz microwave ablation at 16 W for 45–160 s, and the target temperature was 80°C; eight patients received a target temperature of 90°C and microwave ablation for 4–6 min^13^, ^14^, ^23^, ^24^, ^25^, ^26^.

In the clinical application, the specific working parameters of the equipment to be used should be referred to, aimed at the target lesions of different sizes and locations. Coagulative tumor necrosis should be considered as the ablation target.

## Recommendation 6: Tumor Nest Ablation is the Target of Microwave Therapy for Osteoid Osteoma in Extremities, and Pain Improvement is the Evaluation Standard

Pain is the most common clinical symptom of osteoid osteoma in extremities, and less than 2% of patients show no pain^22^. Pain scoring is a common and simple method for evaluating the clinical effect of microwave ablation of osteoid osteoma^13^, ^14^, ^24^, ^25^, ^26^. The change in MRI before and after microwave ablation is also regarded as the objective basis for the imaging evaluation of microwave ablation. Quantitative MRI perfusion analysis can objectively evaluate the therapeutic effect of percutaneous microwave ablation on osteoid osteoma. Plasma flow and volume distribution may be reliable indicators of successful thermal ablation^23^, ^24^, ^25^.

Treatment of osteoid osteoma aims to eliminate pain by percutaneous microwave ablation. The effect of the treatment can be feasibly evaluated by improving pain. Changes in MRI before and after treatment can be used as an objective evaluation basis for percutaneous microwave ablation to treat osteoid osteoma in extremities.

## Recommendation 7: The Clinical Effect of Intraoperative Microwave Ablation is Influenced by Tumor Size, Texture, Blood Supply, Surgeon Experience, and Other Factors

The thermal effect of microwave ablation is closely related to the type of substance in the tumor. The difference in water content in various substances can lead to differences in temperature rise and microwave absorption^10^, ^39^. Therefore, microwave ablation exerts an unpredictable effect on a tumor with a complex component structure and a large body. During microwave ablation of the thermal tumor, the tumor content constantly changes in uneven real time, such as coagulation and necrosis, resulting in a continuous change in the structure of the microwave field in the tissue, which is difficult to accurately evaluate using a mathematical model^40^. Intraoperative microwave ablation has been widely used in the treatment of benign and malignant tumors in extremities, including osteosarcoma, Ewing's sarcoma, malignant fibrous histiocytoma, chondrosarcoma, soft tissue sarcoma, giant cell tumor of bone, metastatic cancer of the bone, and so on^18^, ^41^, ^42^, ^43^, ^44^, ^45^.

The clinical effect of microwave ablation in the treatment of bone tumors in extremities can be effectively improved by comprehensively considering the tumor (benign and malignant), volume size, internal material composition, and site characteristics; flexibly arranging needles; and setting power to different intensities for microwave ablation to achieve complete ablation of tumors and retention of the bone structure.

## Recommendation 8: *In Situ* Ablation is the First Choice for Microwave Ablation of Bone Tumors in Extremities

Microwave ablation of tumors varies from other ablation methods in that it changes tumor cells into heat sources by using the electromagnetic field, and thermal ablation starts from the inside of the tumor^39^, ^46^. In the clinical application, contrary to *in vitro* inactivation and replantation of bone tumor resection, microwave ablation can achieve tumor inactivation *via in situ* ablation after protecting the normal tissues around bone tumors of the limbs^18^, ^41^.

*In situ* microwave ablation of bone tumors in extremities retains the natural continuity of bone tissue to the greatest extent, which can help reconstruct and remodel bone tissue in the long term and prevent the problem of osteotomy and bone healing that needs to be considered in tumor resection, inactivation, and replantation. The bone may also be cut off on one side to separate the tumor and ablate lesions.

## Recommendation 9: Microwave Ablation of Bone Tumors in Extremities Under Appropriate Temperature and Time is Recommended

Microwave ablation of tumors needs to achieve a certain dose of thermal injury, which is positively correlated to temperature and time. Different tumor cells exhibit different levels of sensitivity to hyperthermia^1^. The results of a hyperthermia study indicate that osteosarcoma cells can be killed by heating at 50°C for 15 min^6^. Animal experiments indicate that heat treatment at 65°C for 30–120 min can inactivate cells and retain the osteogenic characteristics of bone tissue^47^. A biomechanical study on a human normal cortical bone undergoing microwave ablation indicates that microwave ablation at 80°C for 30 min can significantly damage the toughness and plasticity of the cortical bone and increase the brittleness of bone tissue, leading to bone fracture^48^. A biomechanical study of an animal bone that underwent microwave heating indicates a significant decrease in bone strength at 75°C for 30 min but only a slight reduction at 60°C for 30 min^49^. A retrospective study of clinical cases shows that microwave ablation can achieve satisfactory results in the treatment of malignant pelvic and limb bone tumors at 70–80°C ablation temperature for 30 min^50^. Several studies have suggested that ablation *in vivo* is more complex than *in vitro*. Bone tissue protects cells to a certain dose, and some areas elude the effects of hyperthermia^51^. Tumor ablation temperatures exceeding 60°C reportedly achieve the treatment effect in clinical applications^52^, ^53^.

The desired effect of microwave ablation of bone tumors in extremities is to completely kill tumor cells while preserving the biomechanical and biological properties of bone tissue. Regardless of the settings of the microwave ablation equipment, ablation temperature and duration are important factors to ensure the therapeutic effect. Generally, the temperature should be controlled at 60–80°C for 30 min.

## Recommendation 10: For Large‐Sized Bone Tumors in Extremities, Multiple‐Antenna Microwave Ablation May Be Useful

Owing to high frequency, short wavelength, and shallow penetration depth, medical microwave is generally about 3 cm. To achieve complete ablation of large tumors, multiple‐antenna microwave ablation is a safe and effective choice^16^, ^39^, ^41^, ^54^, ^55^, ^56^. Using microwave antenna arrays is regarded as a safe and effective approach to treating malignant or invasive bone tumors in the limb^54^. Multiple‐microwave antennas can also be used to ablate the tumor segment in the matrix mode, with an array spacing of 3 cm between microwave antennas and a spacing of 4 cm for each ablation depth^56^.

When the limb bone has a large tumor body, complete ablation of the whole tumor body is difficult to accomplish with a single needle. Multiple punctures with a single needle may cause tumor cells to spill out and pollute the normal tissue. Therefore, multiple‐needle ablation can be used. These microwave needles can be inserted into the tumor body at multiple angles to avoid the blind spot of ablation.

## Recommendation 11: Microwave Ablation of Bone Tumors with an Abundant Blood Supply Can Reduce Tumor Bleeding During Curettage

Microwave ablation can lead to coagulative necrosis of the tumor tissue, including tumor blood vessels, which is beneficial to the exposure of the surgical field and reduction of bleeding^42^, ^57^, ^58^. In clinical research, microwave ablation can reduce tumor bleeding and intraoperative blood transfusion before bone metastasis curettage and may more effectively occlude blood vessels, compared with radiofrequency treatment^57^. In addition, microwave ablation can cause bone metastasis coagulation and necrosis, block blood vessels, and reduce bleeding to improve the safety of surgery^42^. Another study suggested that microwave ablation of periacetabular bone metastases could reduce intraoperative bleeding^58^.

For bone tumors with an abundant blood supply, a higher ablation temperature can be easily achieved by microwave ablation. Numerous tumor blood vessels are present in an aneurysmal bone cyst, a giant cell tumor of bone, bone metastasis from liver cancer, bone metastasis from kidney cancer, and other tumor lesions. Severe bleeding occurs during curettage of these tumors. Microwave ablation before curettage can significantly reduce tumor bleeding and improve surgical safety.

## Recommendation 12: Microwave Ablation of Skip Metastasis in the Medullary Cavity of Osteosarcoma Helps Retain a Larger Bone Structure

Skip metastasis in the medullary cavity of osteosarcoma has an incidence ranging from 1.4% to 10%^17^, ^59^, ^60^, ^61^, which often indicates poor prognosis^17^, ^61^. After resection of osteosarcoma of the distal femur, a microwave ablation needle was placed into the medullary cavity to melt skip metastases in the medullary cavity, and then tumor curettage was performed. Moreover, tumor prosthesis reconstruction was conducted in four cases, and thigh amputation was performed in one case. No local recurrence was found in these five patients; three patients died of distant metastasis, and the remaining two patients survived. This technique prevents total femoral replacement and retains good limb function^17^.

**Fig 1:**
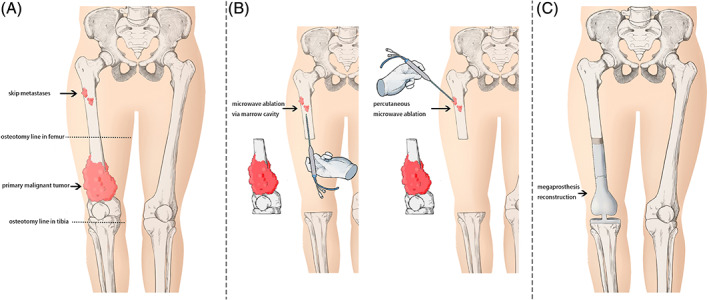
Microwave ablation was used to treat skip metastasis in the proximal femoral medullary cavity of osteosarcoma. (A) Adhere to the principle of limb salvage surgery of malignant tumors, the osteosarcoma in the distal femur was resected. (B) After resection of osteosarcoma in the distal femur, a microwave ablation needle was inserted into the skip metastasis percutaneous or *via* medullary cavity to melt skip metastases, and then tumor curettage was performed. (C) Normal mega‐prosthesis was used to reconstruct the limb function without total femur replacement.

The proximal femur is the most frequent site of osteosarcoma skip metastasis in the medullary cavity. Extensive removal of skip metastases can cause the loss of the long bone in the proximal femur or even the entire femur. Microwave ablation of osteosarcoma skip metastases in the medullary cavity is an alternative treatment with the advantage of retaining a larger portion of the bone in the proximal femur (Fig. 1).

## Recommendation 13: Before Microwave Ablation of Bone Tumors in Extremities, Important Blood Vessels, Nerves, and Soft Tissues Should be Dissociated and Protected

A copper mesh laid on the surrounding soft tissues has been reported to prevent microwaves from passing through during microwave ablation of bone tumors in extremities. However, the blood that accumulates between the tumor and the copper mesh, which can cause scalding of soft tissues, needs prompt removal^41^. The cooling bag is used to isolate the tumor and the surrounding soft tissue, and the cooling protection can be realized by allowing low‐temperature saline to flow through the cooling bag^54^. Several studies have proposed that the fingers of surgeons should be placed on the surface of important structures to roughly detect the tissue temperature and to drip low‐temperature saline on the tissue surface at any time to prevent overheating^55^. A common and simple approach to isolating tumor and the surrounding soft tissue is to use a gauze soaked in cooling saline after tumor separation. Continuous use of hypothermic saline can also protect the surrounding soft tissue during ablation^16^, ^42^, ^55^, ^62^. When the tumor is adjacent to the articular surface, circulating cooling saline can be used to cool the articular cavity during microwave ablation to avoid thermal injury of the articular surface cartilage^16^, ^18^, ^56^.

Before microwave ablation of bone tumors in extremities is conducted, the surrounding large blood vessels, important nerves, and normal tissues need to be protected. During ablation, the target focus can meet the requirements of thermal ablation temperature and avoid scalding of surrounding normal tissues, which can effectively reduce complications related to microwave ablation.

## Recommendation 14: During Microwave Ablation of Bone Tumors In Extremities, Real‐Time Multipoint Temperature Measurement on the Tumor Body, Tumor Side, and Surrounding Tissues Has to be Performed

During microwave ablation of tumors, the temperatures of the tumor surface and of the surrounding normal tissue have to be measured to provide a reference for the evaluation of the effect of tumor ablation and the safety level for the protection of the surrounding tissue^63^, ^64^, ^65^. In the microwave ablation of tumors, invasive thermometry includes thermocouple and thermistor thermometry, whereas non‐invasive thermometry includes electrical impedance thermometry, microwave thermometry, and nuclear magnetic resonance thermometry^64^, ^65^. Invasive thermometry has the following advantages: high response rate, accuracy and reliability, small error (±0.3°C), high resolution (≤0.2°C), and relatively small electromagnetic and thermal interference; its disadvantages include increased trauma during percutaneous use. Meanwhile, noninvasive thermometry fails to reflect the overall equilibrium temperature and exhibits inadequate accuracy^64^, ^65^.

Temperature measurement using a thermocouple or a thermistor can meet the accuracy requirements of microwave ablation but can only reflect the temperature of a specific measurement point. When the tumor is large, multiple thermometers and multiple‐point thermometry can help find the possible blind spots of low‐temperature ablation during microwave ablation, and real‐time thermometry of the surrounding tissue can help promptly reduce the temperature of the surrounding tissue during surgery to avoid accidental scalding.

## Recommendation 15: The Necrotic Tissue Around the Bone Structure Should be Cleaned After Microwave Ablation of Bone Tumors in Extremities

Microwave ablation of bone tumors in extremities leads to the coagulation and necrosis of the tumor tissue. During surgery, the inactive tissue outside the bone contour needs to be removed, the natural shape of the bone has to be restored, and the necrotic tumor tissue in the bone tissue has to be thoroughly cleaned up by opening windows at the bone destruction site and/or the bone surface^19^, ^42^, ^50^, ^66^. Some studies have suggested that after microwave ablation of bone tumors in extremities, the soft tissue attached to the tumor bones should be removed and scraped off, and the sclerotic tumor bone tissue should be reserved for the reconstruction of functional limb^41^, ^56^. A report has identified that inadequate clearance of necrotic tissue is a potential cause of infection after microwave ablation of bone tumors in extremities^66^.

After microwave ablation of bone tumors in extremities, the coagulative necrotic soft tissue attached outside the bone should be removed to expose the bony structure. The necrotic tumor tissue in the bone should be thoroughly cleaned up. The fragmentary tissue around the bony structure should also be cleaned up to restore the natural shape of the bone for reconstruction of the functional limb.

## Recommendation 16: After Microwave Ablation of Bone Tumors in Extremities, Appropriate Reconstruction Should be Selected Depending on the Nature, Location, and Type of Bone Defects

After microwave ablation of bone tumors in extremities, bone defects usually require proper repair and reconstruction. In some studies, an autogenous fibula graft has been used to repair bone defects after microwave ablation of primary malignant bone tumors in extremities, and autogenous bone graft or allogeneic bone graft mixed with bone cement is used to repair and reconstruct the remaining cavities. This technique can achieve bone tissue regeneration and biological reconstruction^18^, ^54^, ^67^, ^68^. Biological reconstruction has to be focused on the repair time of bone tissue. Results of animal experiments indicate that bone revascularization and regeneration occur 6 months after microwave ablation, and the dead bone area still comprises more than 50% within 1 year after surgery^69^. Histopathological examination of osteosarcoma in the distal femur showed that 43 months after microwave ablation, necrosis remained, revascularization was not completed, and risk of fracture was still present^70^. Bone cement can be used to repair bone defects after microwave ablation of bone metastases in extremities. This method is simple and practical with satisfactory clinical effects^42^. The immediate stability of a tumor cavity filled with bone cement contributes to the early recovery of limb function, but bone cement filling adjacent to the articular surface may lead to painless arthritis^71^. In addition, compared with the dynamic process of bone graft reconstruction, the relatively static environment after bone cement filling is more conducive to postoperative imaging follow‐up observation^71^, ^72^.

In the reconstruction of bone defects after microwave ablation of bone tumors in extremities, appropriate methods should be selected depending on the type of bone defect in extremities. Repair of bone defects by bone cement filling is conducive to early weight‐bearing and functional rehabilitation; however, long‐term degeneration of adjacent bone joints is possible.

## Recommendation 17: Appropriate Internal Fixation is Recommended After Microwave Ablation of Bone Tumors in Extremities

Microwave ablation of bone tumors in extremities can inevitably reduce the biological activity and mechanical strength of the remaining bone^48^, ^49^. Revascularization and reconstruction of the bone tissue require a long time^69^, ^70^. Bone strength needs to be evaluated prior to surgery of bone tumors in extremities^73^. Depending on the bone strength of extremities, extent of bone defect for repair and reconstruction, and the specific situation during surgery, internal fixation may be necessary to strengthen the bone strength of the load‐bearing bones in extremities. Some studies show that regardless of the presence of osteotomy after the completion of bone defect repair and reconstruction, microwave ablation of malignant bone tumors in extremities requires plate‐and‐screw internal fixation to strengthen the inactivated bone segment and prevent a pathologic fracture^50^. Intramedullary fixation after microwave ablation can also prevent pathological fractures^56^. Three‐dimensional printing has also been recently used to produce a titanium plate with a personalized bone structure, which is fixed after microwave ablation of a tumor around the knee joint. Such customized plate fixation reportedly reduces the occurrence of a pathological fracture and improves the limb function of patients^74^.

After microwave ablation of bone tumors in extremities, using bone plates for internal fixation provides ease of operation and effective fixation. However, for bone ablation without osteotomy and bone defect, the intramedullary nail can also be used.

## Recommendation 18: Pathological Fracture is a Common Complication of Microwave Ablation of Bone Tumors in Extremities

Complications after microwave ablation of bone tumors in extremities rarely occur, with a total incidence of 12.9%–73.3%. Pathological fracture is the most common complication with an incidence of 2.6%–13.3%^18^, ^41^, ^56^, ^66^, ^75^. In a retrospective study of 89 patients with bone tumors in extremities that underwent microwave ablation, pathological fracture occurred in five patients with tumors in the lower femur after 16.8 months on average^41^. In a clinical study involving 15 patients with osteosarcoma in the lower extremities who received microwave ablation, pathological fracture occurred in six patients after 20.8 months on the average^56^. Another report showed that out of 54 patients with bone tumors in extremities who underwent microwave ablation, four patients exhibited pathological fracture 6–12 months after surgery^66^. Meanwhile, a pathological fracture was reported 8–16 months after surgery in five of 38 patients with malignant bone tumors in extremities^75^.

Unlike the ablation of other organ tumors, microwave ablation of bone tumors needs to consider complete ablation of tumors and protection of the surrounding tissue, as well as the osteogenic ability and biological strength of bone preservation. A long period of bone reconstruction is needed after microwave ablation. Rigid internal fixation and extension of the unloading bearing time of the affected limb can help reduce the occurrence of pathological fracture.

## Recommendation 19: Microwave Ablation of Primary Malignant High‐Grade Bone Tumors in Extremities Requires the Inactivation of All Tumor Cells

For primary malignant high‐grade bone tumors in extremities undergoing microwave ablation, the recurrence rate is 7.9%–26.7%^18^, ^41^, ^56^, ^66^, ^75^. In a retrospective study involving 469 patients with malignant bone tumors in extremities, 46 patients experienced recurrence—nine patients who underwent limb salvage surgery and 35 patients who underwent amputation^18^. A clinical report involving 81 patients with malignant bone tumors in extremities revealed recurrence originating from the soft tissue in eight patients, among which six underwent reoperation, as determined by postoperative pathological analysis^41^. Research on 15 patients who received microwave ablation of osteosarcoma in the lower extremities showed recurrence in four patients with metaphyseal osteosarcoma—three cases in the bone and one case in the soft tissue^56^. Microwave ablation of 38 patients with malignant bone tumors in extremities resulted in recurrence in three patients, with one case related to inappropriate preoperative biopsy^75^.

The recurrence rates of primary malignant high‐grade bone tumors in extremities after microwave ablation vary, which is related to the lack of standardized operating procedures. The effect of ablation often depends on the personal experience of the surgeon and the irregular shape of tumors, impeding the complete inactivation of tumor cells. Complete inactivation of tumor cells should be prioritized in clinical applications.

## Recommendation 20: Microwave Ablation Can be Used in Suspected Areas Outside Bone Tumors in Extremities

Limb salvage surgery for malignant bone tumors in extremities should first ensure local control of the tumor, which is achieved with a safe surgical boundary as the most important factor^76^, ^77^. Limb salvage surgery for malignant bone tumors in extremities should adopt microwave ablation as an adjuvant therapy. Before the microwave ablation needle is inserted, the principle of tumor resection should be followed to thoroughly expose the local tumor and isolate the tumor from the surrounding normal soft tissue^18^, ^41^, ^50^, ^56^, ^62^, ^67^. Microwave ablation was conducted to treat suspected areas in 11 cases of malignant bone tumors in extremities that show good response to chemotherapy near the articular surface. No histopathological analysis of the ablation area was conducted; regardless, no local tumor recurrence was reported in the follow‐up of more than 3 years after surgery. Microwave ablation can effectively enhance the safety of the operation boundary^78^.

Microwave ablation of bone tumors in extremities is a limb salvage technique, which should adhere to the principle of limb salvage surgery of malignant tumors. The principle of tumor surgery should be followed in the process of exposure. For malignant bone tumors that respond well to chemotherapy, microwave ablation can be used to ablate the suspected area to preserve the important bone structure under special circumstances. Notably, the actual surgery is difficult, and skilled surgical techniques, rather than conventional techniques, are required for bone tumors.

## Recommendation 21: Selection of Appropriate Microwave Ablation Equipment Can Facilitate Intraoperative Ablation of Bone Tumors

The medical microwave ablation equipment in China is designed as a needle structure, with round and pointed designs. Most equipment has a power of 2450 MHz, whereas few devices have a power of 915 MHz. The multiple‐source host can be used by multiple microwave needles simultaneously.

With the Chinese microwave ablation needle as an example, bone tumor ablation can be conducted using a microwave cold circulation needle measuring 2.0–3.2 mm in diameter and 150–200 mm in length. The microwave antenna adopts a vertical slot antenna structure, with an ablation range of 54 × 42 mm, and the maximum parameters of a single‐electrode bearing are 120 W and 15 min.

## Recommendation 22: The Use of a Microwave Ablation Needle with Temperature Monitoring Can Reduce the Complications of Percutaneous Microwave Ablation of Bone Tumors in Extremities

The development of a microwave needle has undergone three generations. The first generation of the microwave ablation needle radiator is at the tip, which can be easily damaged during puncture. The radiator has no cooling device, resulting in a high needle temperature. The needle temperature can reach 60°C at most, which can easily burn the skin. In 2002, the second generation of the microwave ablation needle, referred to as the water‐cooled microwave ablation needle, was developed in China. The built‐in cooling system device can reduce the needle temperature when the microwave energy is converted in the tumor, reduce skin scalding and carbonization at the core of the ablation range, cause “trailing” to disappear, and improve the form of ablation. The third‐generation intelligent monitoring microwave ablation needle provides big data real‐time ablation data through the software system with the output power of a real‐time monitoring ablation needle.

The needle has to penetrate the skin, subcutaneous tissue, and muscle tissue in percutaneous microwave ablation. Using a microwave ablation needle with temperature monitoring can improve the safety of operation and reduce the scalding of the soft tissue.
